# Rabies control in KwaZulu-Natal, South Africa

**DOI:** 10.2471/BLT.17.194886

**Published:** 2018-04-12

**Authors:** K LeRoux, D Stewart, KD Perrett, LH Nel, JA Kessels, B Abela-Ridder

**Affiliations:** a Allerton Provincial Veterinary Laboratory, KwaZulu-Natal Department of Environment, Agriculture and Rural Development, Pietermaritzburg, South Africa.; b Wild Capture CC, Drummond, KwaZulu-Natal, South Africa.; c Veterinary Services, KwaZulu-Natal Department of Environment, Agriculture and Rural Development, Pietermaritzburg, South Africa.; d Faculty of Natural and Agricultural Sciences, University of Pretoria, Pretoria, South Africa.; e School of Veterinary Science, University of Queensland, Building 8114, Inner Ring Road, Gatton, Queensland, 4343, Australia.; f Neglected Zoonotic Diseases, World Health Organization, Geneva, Switzerland.

## Abstract

**Problem:**

Urbanization, large dog populations and failed control efforts have contributed to continuing endemicity of dog-mediated rabies in KwaZulu-Natal province, South Africa.

**Approach:**

From 2007 to 2014 we used a OneHealth approach to rabies prevention, involving both the human and animal health sectors. We implemented mass vaccination campaigns for dogs to control canine rabies, and strategies to improve rabies awareness and access to postexposure prophylaxis for people exposed to rabies.

**Local setting:**

A rabies-endemic region, KwaZulu-Natal is one of the smallest and most populous South African provinces (estimated population 10 900 000). Canine rabies has persisted since its introduction in 1976, causing an average of 9.2 human rabies cases per annum in KwaZulu-Natal from 1976 to 2007, when the project started.

**Relevant changes:**

Between 2007 and 2014, the numbers of dog vaccinations rose from 358 611 to 395 000 and human vaccines purchased increased form 100 046 to 156 996. Strategic dog vaccination successfully reduced rabies transmission within dog populations, reducing canine rabies cases from 473 in 2007 to 37 in 2014. Actions taken to reduce the incidence of canine rabies, increase public awareness of rabies and improve delivery of postexposure prophylaxis contributed to reaching zero human rabies cases in KwaZulu-Natal in 2014.

**Lessons learnt:**

Starting small and scaling up enabled us to build strategies that fitted various local settings and to successfully apply a OneHealth approach. Important to the success of the project were employing competent, motivated staff, and providing resources, training and support for field workers.

## Résumé

**Problème:**

L'urbanisation, les importantes populations canines et les mesures de lutte inefficaces ont contribué à l'endémicité persistante de la rage transmise par les chiens dans la province du KwaZulu-Natal, en Afrique du Sud.

**Approche:**

De 2007 à 2014, nous avons appliqué l'approche «Un monde, une santé» pour la prévention de la rage, et mobilisé les secteurs de la santé humaine et de la santé animale. Nous avons mené des campagnes de vaccination massive des chiens en vue de lutter contre la rage canine, et déployé des stratégies pour mieux sensibiliser la population à la rage et améliorer l'accès des personnes exposées à la rage à la prophylaxie post-exposition.

**Environnement local:**

Le KwaZulu-Natal, où la rage est endémique, est l'une des provinces d'Afrique du Sud les plus petites et les plus peuplées (population estimée à 10 900 000 habitants). La rage canine persiste depuis son apparition en 1976, ayant provoqué en moyenne 9,2 cas de rage humaine par an au KwaZulu-Natal entre 1976 et 2007, année qui a marqué le lancement du projet.

**Changements significatifs:**

Entre 2007 et 2014, le nombre de vaccinations pratiquées sur des chiens est passé de 358 611 à 395 000, tandis que le nombre de vaccins humains achetés est passé de 100 046 à 156 996. La vaccination stratégique des chiens a permis de faire reculer la transmission de la rage au sein des populations canines, le nombre de cas de rage animale passant de 473 en 2007 à 37 en 2014. Les actions prises pour réduire l'incidence de la rage canine, sensibiliser la population à la rage et améliorer l'accès à la prophylaxie post-exposition ont permis qu’aucun cas de rage humaine ne soit déclaré au KwaZulu-Natal en 2014.

**Leçons tirées:**

En démarrant notre projet sur une petite échelle, puis en lui donnant une ampleur accrue, nous avons pu élaborer des stratégies adaptées aux différents contextes locaux et appliquer avec succès l'approche «Un monde, une santé». Le succès du projet a reposé en grande partie sur le recrutement de professionnels compétents et motivés, et la mise à disposition de ressources, de formations et d'un soutien pour les agents locaux.

## Resumen

**Problema:**

La urbanización, las grandes poblaciones de perros y los esfuerzos fallidos de control han contribuido a la continuación de la transmisión de la rabia por perros en la provincia de KwaZulu-Natal, Sudáfrica.

**Enfoque:**

De 2007 a 2014, se utilizó un enfoque «Una salud» para la prevención de la rabia, que involucraba a los sectores de salud humana y animal. Se implementaron campañas de vacunación masiva para perros para controlar la rabia canina, así como estrategias para mejorar la concienciación sobre la rabia y el acceso a la profilaxis posterior a la exposición para las personas expuestas a la rabia.

**Marco regional:**

Una región endémica de la rabia, KwaZulu-Natal es una de las provincias sudafricanas más pequeñas y pobladas (población estimada en 10 900 000). La rabia canina ha persistido desde su aparición en 1976, causando una media de 9,2 casos de rabia en humanos al año en KwaZulu-Natal entre 1976 y 2007, cuando comenzó el proyecto.

**Cambios importantes:**

Entre 2007 y 2014, el número de vacunas administradas a perros aumentó de 358 611 a 395 000, mientras que las vacunas humanas adquiridas aumentaron de 100 046 a 156 996. La vacunación estratégica en los perros redujo con éxito la transmisión de la rabia en las poblaciones caninas, lo que redujo los casos de rabia animal de 473 en 2007 a 37 en 2014. Las medidas adoptadas para reducir la incidencia de la rabia canina, aumentar la concienciación pública sobre la rabia y mejorar el suministro de profilaxis posterior a la exposición contribuyeron a que en KwaZulu-Natal no se dieran casos de rabia humana en 2014.

**Lecciones aprendidas:**

Empezar a pequeña escala y después incrementar los esfuerzos permitió crear estrategias que se ajustasen a los diversos marcos regionales e implementar con éxito un enfoque «Una salud». Para que el proyecto tuviera éxito, fue importante contar con personal competente y motivado, así como ofrecer recursos, formación y apoyo a los trabajadores de campo.

## ملخص

**المشكلة:**

أسهمت عوامل التوسّع الحضري، وتزايد أعداد الكلاب، والقصور في جهود الرقابة إلى استمرار استشراء حالات الإصابة بداء الكلب المنقولة عبر الكلاب في إقليم كوازولو ناتال بجنوب أفريقيا.

**الأسلوب:**

في الفترة ما بين عاميّ 2007 وحتى 2014، استخدمنا نهج "توحيد الأداء في مجال الصحة" للوقاية من داء الكلب، والذي انطوى على قطاعيّ الصحة البشرية والحيوانية. وقمنا بتنفيذ حملات تطعيم واسعة النطاق للكلاب للسيطرة على حالات الإصابة بداء الكلب بين الحيوانات من فصيلة الكلبيات، كما عمدنا إلى تطبيق استراتيجيات تهدف إلى تحسين مستوى الوعي بداء الكلب وتيسير سبل الوصول إلى الوقاية الطبية بعد التعرض إلى المرض وذلك بالنسبة للأشخاص الذين تعرضوا إلى داء الكلب.

**المواقع المحلية:**

تمثل كوازولو ناتال منطقة يستشري فيها داء الكلب، وهي تندرج بين الأقاليم الأصغر حجمًا والأعلى كثافة سكانية في جنوب أفريقيا (إذ تشير تقديرات عدد السكان إلى وجود 10 ملايين وتسعمائة ألف نسمة). وقد استمر انتشار حالات الإصابة بداء الكلب المنقول عن الحيوانات من فصيلة الكلبيات منذ ظهوره في عام 1976، مسببًا حالات من الإصابة تنتشر بواقع 9.2 حالة إصابة بين البشر في العام الواحد في إقليم كوازولو ناتال، وذلك في الفترة ما بين عاميّ 1976 وحتى 2007 وهي الفترة التي بدأ فيها المشروع.

**التغيّرات ذات الصلة:**

في الفترة ما بين عاميّ 2007 و2014، ارتفعت أعداد عمليات تطعيم الكلاب من 358,611 إلى 395,000، كما زاد عدد التطعيمات البشرية التي تم شراؤها من 100,046 إلى 156,996. ونجح التطعيم الاستراتيجي للكلاب في الحد من حالات انتشار داء الكلب بين أعداد الكلاب، فأدى بذلك إلى تناقص حالات الإصابة بداء الكلب بين الحيوانات من 473 حالة في عام 2007 إلى 37 حالة في عام 2014. وقد أسهمت الإجراءات الموجهة لتقليل حالات الإصابة بداء الكلب بين الحيوانات من فصلية الكلبيات، وزيادة الوعي الجماهيري بالداء، وتحسين خدمات تقديم الوقاية الطبية عقب التعرض إلى المرض أسهمت في القضاء على حالات الإصابة بداء الكلب بين البشر في كوازولو ناتال عام 2014.

**الدروس المستفادة:**

قادتنا البداية المتواضعة مع تزايد الجهود تدريجيًا إلى وضع استراتيجيات تتناسب مع المواقع المحلية، وتطبيق نهج "توحيد الأداء في مجال الصحة" بنجاح. ومن بين العوامل المهمة في نجاح المشروع، كان هناك اللجوء إلى توظيف فريق عمل كفوء ولديه الحافز للعمل، فضلاً عن توفير الموارد اللازمة والتدريب والدعم للعاملين الميدانيين.

## 摘要

**问题:**

城市化进程、大量的狗群以及无效的控制工作导致犬类介导的狂犬病在南非共和国夸祖鲁纳塔尔省内持续流行。

**方法:**

2007 年至 2014 年，我们使用了同一健康 (OneHealth) 方法研究狂犬病预防，其涉及人类和动物健康领域。为了控制狗群中的狂犬病，我们实施了大规模疫苗接种运动，同时采取了一些策略来提高公众对狂犬病的认识并为接触狂犬病的人群提供接触后的预防措施。

**当地状况:**

狂犬病流行区——夸祖鲁纳塔尔省是南非共和国面积最小、人口最多的省份之一（估计人口为 10 900 000）。自 1976 年传入以来，狂犬病一直持续存在。1976 年至 2007 年本项目开始，夸祖鲁纳塔尔省每年平均发生的人类狂犬病病例为 9.2 例。

**相关变化:**

2007 年至 2014 年期间，狗接种疫苗数量从 358 611 上升至 395 000，购买的人类疫苗数量从 100 046 上升至 156 996。战略性的狗接种疫苗成功减少了狗群中的狂犬病传播，并将动物狂犬病病例从 2007 年的 473 例降至 2014 年的 37 例。采取行动以降低狂犬病的发病率、提高公众对狂犬病的认识和改进接触后的预防措施，有助于 2014 年夸祖鲁纳塔尔省实现的零人类狂犬病病例。

**经验教训:**

从小规模开始并扩大规模，使我们能够制定符合当地各种环境的策略，并成功应用同一健康 (OneHealth) 方法。该项目成功的关键在于雇用出色且积极的工作人员，并为实地工作人员提供资源、培训和支持。****

## Резюме

**Проблема:**

Урбанизация, большие популяции собак и неудачные попытки контроля способствовали сохранению эндемичности по бешенству, переносимому собаками, в провинции Квазулу-Наталь, Южная Африка.

**Подход:**

С 2007 по 2014 год авторы использовали подход OneHealth к профилактике бешенства, включающий секторы здравоохранения как людей, так и животных. В рамках борьбы с бешенством авторы провели массовые кампании по вакцинации собак, а также мероприятия по повышению осведомленности о бешенстве и улучшению доступа к постконтактной профилактике для людей, подвергшихся воздействию вируса бешенства.

**Местные условия:**

Эндемичный по бешенству район Квазулу-Наталь — одна из самых маленьких и густонаселенных южноафриканских провинций (численность населения составляет 10 900 000 человек). Ситуация по бешенству собак, которая сохраняется с 1976 года, приводит в среднем к 9,2 случая заболевания человека бешенством в год в Квазулу-Натале в период с 1976 по 2007 год (с начала действия проекта).

**Осуществленные перемены:**

В период с 2007 по 2014 год количество проведенных вакцинаций для собак возросло с 358 611 до 395 000, а количество закупленных вакцин для людей увеличилось с 100 046 до 156 996. Стратегическая вакцинация собак успешно сократила передачу бешенства внутри популяций собак, сократив количество случаев бешенства у животных с 473 в 2007 году до 37 в 2014 году. Меры, принятые для снижения заболеваемости бешенством у собак, повышения осведомленности общественности о бешенстве и оптимизации проведения постконтактной профилактики, способствовали достижению нулевых случаев заболевания бешенством у людей в Квазулу-Натале в 2014 году.

**Выводы:**

Начав с малого и постепенно увеличивая масштабы, авторы создали стратегии, которые подходят для различных местных условий, и успешно применяли подход OneHealth. Привлечение компетентного, мотивированного персонала и предоставление ресурсов, обучения и поддержки работникам на местах были важными аспектами для успеха проекта.

## Introduction

Rabies is a fatal zoonotic disease, causing tens of thousands of human deaths each year.^1^ Most human rabies cases worldwide are caused by dog bites^2^ and are preventable through canine vaccination and the provision of rabies postexposure prophylaxis to exposed persons.^3^^,^^4^ Many rabies-endemic countries lack the finances and infrastructure to sustainably vaccinate dogs, conduct surveillance or provide communities with access to rabies vaccines.

Vaccinating 70% of the canine population is currently recommended to interrupt transmission of rabies.^5^^–^^7^ However, the scale of this task can be a deterrent to setting up control programmes. Furthermore, quality baseline data on dog populations are viewed as necessary to target canine vaccination campaigns effectively and to measure vaccination coverage accurately.^8^ In many rabies-endemic countries reliable data are scarce.^1^

People exposed to rabies require timely prophylaxis including wound cleaning, vaccines and sometimes rabies immunoglobulins. Effective delivery of postexposure prophylaxis relies on good public awareness of rabies, and access to treatment.

Canine rabies has persisted in KwaZulu-Natal province of South Africa since its introduction in 1976.^9^ Since then, urbanization, large dog populations and failed control efforts have contributed to its endemic status. We describe the KwaZulu-Natal rabies project from 2007‒2014, established to eliminate human rabies through control of canine rabies and to design a programme that could be rolled out in neighbouring regions and countries.

## Local setting

Located on the eastern seaboard of South Africa, KwaZulu-Natal province has an estimated human population 10 900 000.^10^^,^^11^ In 2007, the year the project started, 473 cases of animal rabies were reported. The numbers of dog vaccinations done and human vaccines purchased the same year were 358 611 and 100 046, respectively. Human rabies vaccines and rabies immunoglobulins are provided free of charge in KwaZulu-Natal and the relatively small annual number of human deaths in KwaZulu-Natal (mean 9.2 over the period 1976 to 2007) was attributed to effective use, and overuse, of expensive intramuscular postexposure prophylaxis.

## Approach

The project used a OneHealth approach to human rabies control, involving both the human and animal health sectors in mass vaccination of dogs, public education and awareness, and improved delivery of rabies postexposure prophylaxis. Staff members of the KwaZulu-Natal Department of Environment, Agriculture and Rural Development coordinated the programme, with funding from the Bill and Melinda Gates Foundation. The programme was managed via the World Health Organization. There was collaboration with animal welfare groups, academics, nongovernmental organizations and the human health-care sector through rabies action groups and trainings held for health professionals. We started in one village in 2007, later scaling up the programme to the entire province, and eventually neighbouring provinces and countries.

The first objective was to collect or estimate baseline data on dog populations. To understand the types of communities at risk of rabies we obtained national human census data, supported by disease data and local knowledge of the area. Additionally, we supported a study of local dog ecology to better understand canine populations, dog‒human interactions and factors preventing access to dogs for vaccination.^12^ This used surveys and community interviews to identify high-risk characteristics of canine populations, and dog ownership and management related to rabies endemicity in the province. We also created a database to record laboratory-confirmed human and canine rabies cases and used historic and current rabies data to inform campaign planning.

We initially measured canine rabies vaccination coverage in two villages of KwaZulu-Natal in a census to estimate the dog population. Once the effectiveness of our vaccination strategy was established, we did not expend further resources to evaluate this and thereafter we used rabies incidence in dogs and people as an indicator of the success of the project.

The second objective was to improve the systems for treating exposed people. Awareness of rabies in KwaZulu-Natal was already high,^12^ but we aimed to improve the public’s awareness of rabies, and to encourage them to seek treatment after a suspected exposure. We did this through rabies action groups and locally produced pamphlets, posters, radio broadcasts and newspaper advertisements directed towards medical professionals and the public. To avoid inappropriate use of post exposure treatment we trained medical staff in how to perform risk assessments of exposed patients.^4^ Among the different training interventions were orientations for new staff (conducted by hospitals throughout the province) on assessing and prioritizing animal-bite patients, supported by the materials generated by the project for health-care workers on managing dog bites.

The third objective was to improve the control of rabies in domestic dogs. We initially used sterilization of domestic dogs for canine population control. However, this proved slow and expensive, with little overall impact on dog population size. This is consistent with reports that rabies control can be achieved without population management.^13^^,^^14^ Instead, we used existing knowledge of the local rabies epidemiology to implement targeted vaccination campaigns. For example, we vaccinated dogs in potential source areas to halt disease transmission to adjacent areas. This approach was informed by project staff who understood the local conditions and by sound surveillance systems that identified areas where rabies was more prevalent.

To conduct the vaccination campaigns, we trained and equipped technicians to use humane animal-handling equipment to catch and handle dogs. Printed and broadcast media informed the public on why, how and where to participate. We equipped field workers with vehicles and public address systems allowed staff to call the public to the nearest road to have their dogs vaccinated. By reducing travel distances for owners, we aimed to improve turnout and allow technicians to follow up on animals that were easier to handle at home. We equipped vaccinators to catch and restrain unmanageable dogs; however, free-roaming dogs that could not be caught were left alone. Finally, we created a canine rabies vaccine bank at the provincial veterinary laboratory to provide stability of vaccine supplies, lower prices for bulk orders and a base for expansion of the project.

The fourth objective was to improve the surveillance and diagnostics of rabies. We aimed to increase the submission of samples from suspect animals for laboratory diagnosis by raising awareness, and improving sample transport systems. The same transport infrastructure required to deliver vaccines to communities was used to transport samples from communities to laboratories for diagnosis.

To complement traditional surveillance methods we also used tools such as typing of viruses and sequencing, to determine transmission histories and enhance our understanding of disease causes and spread.

## Relevant changes

**Fig. 1:**
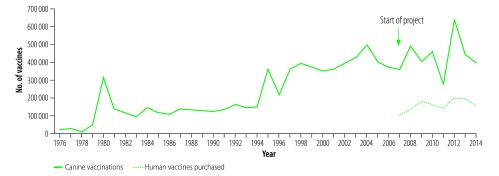
Annual numbers of canine rabies vaccinations done and rabies vaccines purchased for humans in KwaZulu-Natal province, South Africa, 1976–2014

Notes: KwaZulu-Natal province has an estimated human population 10 900 000.^11^ Mass canine vaccination campaigns were implemented in the province from 2007–2014.

**Fig. 2:**
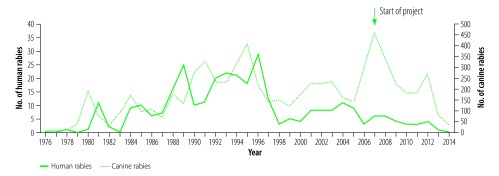
Annual numbers of human and canine rabies cases in KwaZulu-Natal province, South Africa, 1976–2014

Notes: KwaZulu-Natal province has an estimated human population 10 900 000.6 Mass canine vaccination campaigns were implemented in the province from 2007‒2014. The increases in cases in 2012 correspond to interrupted canine vaccination campaigns in 2011.

In 2014, when the project concluded, the annual numbers of dog vaccinations performed and human vaccines purchased in KwaZulu-Natal had increased to 395 000 and 156 996 respectively (Fig. 1). The number of canine rabies cases reported in the province had fallen to 37 and there were no human rabies cases that year (Fig. 2). These figures account only for laboratory-confirmed cases; there were likely additional undiagnosed cases within the province. The increases in cases in 2012 corresponded to interrupted canine vaccination campaigns in 2011 due to an outbreak of foot and mouth disease in the province (diverting attention from dog vaccination campaigns).

## Lessons learnt

We defined success of the project as reaching zero human rabies cases. However, measuring the impact of each intervention is complex. For example, improving the public’s awareness of rabies involved multiple measures over a period of years, and helped to increase turnout in vaccination campaigns, demand for postexposure prophylaxis and submission of samples for surveillance.

We started activities in one area and scaled them up to build systems that fitted the local settings. This allowed rabies awareness, postexposure prophylaxis delivery, dog vaccination and surveillance to improve as the project grew. Local successes generated data, interest and investment, allowed for adaptation, and drove expansion. Effective human management, such as engaging local champions, and training, motivating and equipping field staff, was key to successfully implementing these related interventions (Box 1).

Box 1 **Summary of main lessons learnt**

- Starting small and scaling up enabled us to apply the OneHealth approach successfully and to build strategies that fitted the local setting.
- Important to the success of the project were employing competent, motivated staff, and providing resources, training and support for field workers.
- Our rabies vaccination campaigns generated training materials and standard operating procedures, enabling neighbouring provinces and countries to implement similar campaigns.

Understanding rabies epidemiology within KwaZulu-Natal allowed for targeted interventions in areas where disease transmission was highest. Our experience demonstrates that knowledge and data can be generated while interventions are being implemented; lack of data should not preclude control efforts. 

We found that sustained, targeted, high-coverage dog vaccination in potential source areas consistently halted disease transmission to adjacent areas. Thus, fewer vaccinations were needed to achieve control. The dog vaccination campaigns also generated training materials and standard operating procedures, enabling neighbouring provinces and countries to implement similar campaigns.

In KwaZulu-Natal, high levels of rabies awareness, and established transport systems, including a dedicated courier service and training on transport packaging, contributed to the soundness of the surveillance system. Good surveillance systems are integral to control; however their absence should not preclude implementation of control programmes.

Improving the public’s access to post exposure prophylaxis was likely a factor in reaching zero human rabies deaths in KwaZulu-Natal, as it provides protection even when a low level of disease continues to circulate in dogs. However, we found that increasing the public’s awareness led to increased use of human vaccines, as dog bites continued to occur and be treated as potential exposures even when the incidence of animal rabies was reduced. Therefore, we renewed our focus on bite prevention education. Bite prevention education became a focus of the project towards the end, but was limited by lack of funds, and aims to educate communities on how to interpret dog behaviour to avoid bites. We have yet to evaluate the potential of such education to reduce rabies exposure and demand for postexposure prophylaxis.

Intradermal vaccination would reduce the cost of rabies treatment. However, it is not currently registered in South Africa or included in label recommendations on rabies vaccines. In the long term, the costs of postexposure prophylaxis could be reduced by provision of intradermal vaccines; risk assessments of animal bite patients to prevent unnecessary use of prophylaxis; and bite prevention education to prevent potential rabies exposures.
